# Oxidative Stress and Its Mitigation in Neurodegenerative Disorders

**DOI:** 10.3390/antiox15091190

**Published:** 2026-09-18

**Authors:** David Allan Butterfield

**Affiliations:** 1 Department of Chemistry, University of Kentucky, Lexington, KY 40506, USA; dabcns@uky.edu; 2 Sanders Brown Center on Aging, University of Kentucky, Lexington, KY 40536, USA; 3 Institute of Medicine, University of Maine, Orono, ME 04473, USA; 4 Department of Chemistry, University of Maine, Orono, ME 04473, USA

## 1. Introduction

There is significant evidence that, in neurodegenerative disorders, oxidative stress in the brain is fundamental to the pathophysiology and progression of such conditions, among which is Alzheimer disease (AD) [1]. Oxidative stress is defined as a condition in which the production of free radicals exceeds the free radical scavenging ability of antioxidant proteins or small molecule antioxidants [2,3]. Often in oxidative stress-related neurodegenerative disorders, the diminution of the free radical scavenging function of antioxidant proteins is due to their altered structures following oxidative modification indexed by elevated biomarkers of protein oxidation, lipid peroxidation and/or DNA or RNA oxidation [4,5,6]. Among others, major indices of protein oxidation are elevated levels of protein carbonyls and/or 3-nitrotyrosine [1,2,3,4,5,6,7,8,9,10,11], while major indices of lipid peroxidation are elevated levels of protein-bound 4-hydroxynonenal (HNE) or isoprostanes (from oxidation of lipid chains rich in arachidonic acid) and/or neuroprostanes (from oxidation of lipid chains rich in neuronal-resident docosahexaenoic acid) [1,2,3,11,12,13]. Markers of oxidative damage to DNA or RNA are primarily 8-hydroxy-2-deoxyguanosine or 8-hydroxyguanine, respectively [2,14,15].

Following oxidation of proteins and peroxidation of lipids in AD, the Maillard reaction (aka Amadori chemistry) can result in the accumulation of advanced glycation end products (AGEs) in the AD brain [16]. AGEs assist in the formation of cross-links of both Aβ and tau proteins, hallmarks of AD pathology. Receptors for AGEs (RAGE) are often considered to be an additional biomarker for oxidative damage in AD, and AGE-mediated activation of RAGE is reported to initiate further oxidative damage to neurons [17].

A few represented antioxidant proteins are briefly mentioned here to illustrate that oxidized proteins can contribute to mechanisms of selected neurodegenerative disorders: Cu,Zn-superoxide dismutase, in which genetic or oxidative stress-facilitated altered structure exist are involved in amyotrophic lateral sclerosis (ALS) [18,19]; Mn-superoxide dismutase, alterations of which are involved in certain cancers [20]; and the heme oxygenase-1, biliverdin reductase-A (BVR-A) system, which is involved in AD and Down syndrome (DS) [1,21,22,23]. Another protein that may lose its ability to scavenge the free radical-mediated lipid peroxidation product HNE is apolipoprotein E. This lipoprotein has three isoforms, in which cysteine moieties at residues 112 and 158 in apolipoprotein E2 (which can scavenge HNE) are replaced by arginine (which cannot scavenge HNE) in apolipoprotein E4, and the presence of apolipoprotein E4 is a major risk factor for the development of AD [24,25,26].

In addition to antioxidant proteins, small antioxidant compounds can be either endogenous or exogenous in origin. Certain vitamins, curcumin, ginkgo biloba and ergothioneine are exogenous antioxidants that have varying degrees of efficacy against oxidative damage, as noted below in articles in this Special Issue of *Antioxidants*. Examples of endogenous antioxidants include glutathione, vitamin E, certain flavonoids, selenomethionine, lipoic acid, among others.

This Special Issue of *Antioxidants* on “Oxidative Stress and Its Mitigation in Neurodegenerative Disorders” focuses on the role of oxidative damage in neurodegenerative disorders and how exogenous and endogenous antioxidants or antioxidant proteins mitigate this damage. In 13 papers, investigators delve into the causes and consequences of oxidative stress and, in specific cases, how mitigation of these consequences by use of antioxidant strategies may be potentially beneficial for understanding and treating these neurodegenerative disorders.

## 2. Overview of Published Articles

### 2.1. Oxidative Stress in Selected Neurodegenerative Disorders

#### 2.1.1. Advanced Glycation End Products and Their Receptor in Alzheimer Disease

Professor Patrizia Mecocci and colleagues provide a review paper [Contribution 1] outlining key aspects of advanced glycation end products (AGEs) and their receptor (RAGE) with respect to oxidative stress and neurodegeneration in Alzheimer disease from a metabolic perspective. In particular, the discussion covers many known metabolic processes that are altered in the AD brain following activation of AGE and/or RAGE, including increased amyloidogenic processing of APP, i.e., upregulation of BACE1 to produce more neurotoxic Aβ peptide; decreased mitochondrial-resident apoptosis, i.e., accumulation of cellular detritus; promotion of tau phosphorylation by GSK-3β and MAPKs, which promotes tau aggregation; and disrupted mitochondrial dynamics and function, lessening ATP production and elevating ROS, as a few of many other processes discussed.

Additional discussion of both CNS and peripheral involvement of the AGE-RAGE axis in terms of metabolic disruptions is provided. The review concludes with a brief discussion of potential therapeutic strategies for preventing AGE formation, detoxifying AGE, and inhibiting RAGE signaling in AD.

#### 2.1.2. Proteotoxicity and Neurodegeneration in Down Syndrome

Professor Marzia Perluigi and colleagues comprehensively review the roles of loss of proteostasis functions in early-onset neurodegeneration in Down syndrome (DS), the latter caused by trisomy of chromosome 21 along with transcriptional, metabolic and protein alterations that yield chronic oxidative stress and proteotoxicity [Contribution 2]. The authors review evidence that supports the notion that DS is associated with dysfunction of the proteostasis network, with resulting oxidative stress, which is not prevented by antioxidant response, coupled to endoplasmic reticulum (ER) stress involving protein kinase R-like ER kinase (PERK) signaling and loss of mitochondrial function related to decreased oxidative phosphorylation. Consequent compromise of intracellular degradation systems, including diminution of 26S proteasome activity, ubiquitin-associated signaling decline, and persistent increased activation of mTORC1 that leads to decreased autophagy and mitophagy, causes proteotoxic build-up and consequent neuronal death.

Indeed, persons with DS almost without exception develop neuropathology, including accumulated cellular detritus and the presence of Aβ-rich senile plaques and hyperphosphorylated tau-rich neurofibrillary tangles as well as cognitive decline reminiscent of what is observed in Alzheimer disease. Intriguingly, many of these changes occur in individuals aged 40–50, much earlier than traditional AD. Given the roles played by proteotoxicity in DS and AD, insights into mechanisms of neurodegeneration in both disorders may be gained by deeper examination of proteotoxic mechanisms. Professor Perluigi and her team suggest that therapeutic strategies directed at modifying or preventing the loss of proteostasis and increasing the redox imbalance observed in DS may be a promising strategy for persons with this neurodegenerative disorder.

The author of this Editorial suggests that similar considerations conceivably could be applicable to treatment of AD as well.

#### 2.1.3. Purine DNA Oxidative Damage in Two Neurodegenerative Disorders

After providing an overview of hydroxyl free radical chemistry along with other reactive oxygen species (ROS), Professor Chryssostomos Chatgilialoglu discusses in a review article two neurodegenerative disorders, xeroderma pigmentosum (XP) and Cockayne syndrome (CS) and their relationships to DNA damage from free radical reactions with purines [Contribution 3].

The author describes in detail the chemical reactions involving purine bases of DNA that lead to significant modification of DNA, the protocols for detecting the six possible purine modifications and a discussion of the ineffectiveness of nucleotide excision repair (NER) mechanisms, largely attributable to the steric hindrance caused by modified purines.

Professor Chatgilialoglu describes XP (which has a significant number of subtypes noted as A-G) as an autosomal recessive disorder whose main expressions are skin cancer following UV exposure and in some cases NER impairment is thought to be involved in extensive neurodegenerative damage in patients with XP-A.

Multi-system ineffectiveness, thought to result from NER defects, is characteristic of persons with CS [27]. After describing how nuclear DNA is affected, the author also notes that the products of free radical damage to mitochondrial DNA are different than that of nuclear DNA. Specifically, the lesions involving 8-oxo-purine are far more present than those in nuclear DNA. Base excision repair (BER) is involved in mitochondria, while NER is not present in this organelle.

Further descriptions of XP and CS are found in the current review paper and in the references provided by the author.

### 2.2. Mitigation of Oxidative Stress with Relevance to Neurodegenerative Disorders

#### 2.2.1. Potential Protection Against Parkinson’s Disease by Ergothioneine

Professors Kah-Leong Lim and Barry Halliwell and their colleagues [Contribution 4] review the protective aspects of ergothioneine (ET), a naturally occurring thiol/thione derivative of histidine, for which humans have a high-affinity transporter, organic cation transporter novel type-1 (OCTN1), encoded by the gene *SLC22A4*. However, humans are unable to synthesize ET internally. Diet is the only means of ingestion of ET, and a major dietary source of ET is mushrooms.

The review shows that ET has a high blood–brain barrier penetration capability in marked contrast to curcumin, ginseng, coenzyme Q10 and resveratrol, and ET has a 1-month half-life in the body, a much longer period than most other nutraceuticals. With regard to brain health, several studies, including those from the current authors’ laboratory, demonstrated that lower levels of ET were associated with (1) increased aging parameters; (2) an elevated risk of developing mild cognitive impairment (MCI), an earlier stage of AD; and (3) the lowest levels of ET among patients with dementia [28,29,30].

In PD patients, significantly lower serum or plasma levels of ET were reported [31]. The authors of this review article outline studies that demonstrated in preclinical models of PD, including transgenic mice, *C. elegans* and *Drosophila*, that ET treatment improved survival and rescued various PD-relevant signatures. These PD-relevant indices, which, among others, included damaged mitochondria, elevated oxidative stress, increased levels of α-synuclein, apoptosis, neuroinflammation, decreased glucose metabolism, as well as decreased endoplasmic reticulum signaling, were rescued by ET treatment. Verapamil, an OCTN1 inhibitor, prevented rescue of these PD signatures, consistent with the notion that ET was critically involved in these various rescues of cellular dysfunction in preclinical models of PD.

Professors Lim and Halliwell and their teams review some potential mechanisms by which ET provides protection in neurodegenerative diseases and in aging. The authors suggest that clinical trials of ET in PD are warranted, given the recent positive results demonstrating the safe use of ET in studies of aging and its potential to slow the progression from MCI to AD [30]. The authors note that several questions need to be addressed before ET can be approved as a therapeutic agent for PD or other neurodegenerative disorders. The questions are as follows: (a) Are the lower ET levels observed in persons who demonstrate cognitive decline a cause or consequence of neurodegeneration? (b) What is the therapeutic time frame for ET administration, i.e., how early must ET supplementation be initiated to be effective, and whether benefits remain during late stages of disease? Lastly, which specific mechanism or mechanisms underlie neuroprotection?

#### 2.2.2. Does Ergothioneine Lead to Significant Levels of Potentially Harmful Plasma Trimethylamine-N-Oxide (TMAO) in Humans?

Trimethylamine (TMA) can be formed by breakdown of L-ergothioneine (ET) as well as many other dietary moieties in the gut microbiome. TMA is subsequently oxidized by a liver-resident flavin-containing monooxygenase to form TMAO. The latter compound is associated with increased risk of peripheral deleterious health conditions such as hypertension, atherosclerosis, ischemic stroke, heart failure, myocardial infarction, diabetes, and metabolic syndrome, as well as with neurodegenerative disorders, among which are MCI, AD, and PD [32].

Prof. Barry Halliwell and his team investigated whether ET was a significant contributor to TMAO formation [Contribution 5], since if this were the case, regulatory agencies’ approval of ET use as a therapeutic agent in AD or PD, evidence for which is shown in Contribution 4 as noted above, would be unlikely.

Employing human volunteers, the authors convincingly demonstrate that ET supplementation in a healthy group of Chinese males (25 mg/day for 1 week), as expected, led to ET elevation in plasma and blood; however, there was no significant elevation of mean levels of plasma TMAO. In addition, the authors showed that there was no correlation between levels of ET and levels of TMA or TMAO in plasma of individuals with heart failure (HF). In addition, plasma levels of TMAO, in contrast to ET levels, were elevated in HF, demonstrating a lack of correlation between ET and TMAO.

Given that ET has the status of “Generally Recognized as Safe” by the USA Food and Drug Administration, the results of this study are consistent with the notion that significant levels of TMAO are not generated by ET use and therefore ET is a promising potential neuroprotective therapy for AD and PD.

#### 2.2.3. Short-Chain Fatty Acids Enhance EAAT2-Mediated Glutamate Clearance and Alleviate Oxidative Stress in a MPTP Mouse Model of Parkinson’s Disease

Professor Handeng Liu and co-workers report results of experiments performed to better understand a major mechanism by which oxidative stress occurs in a mouse model of Parkinson disease (PD) [Contribution 6]. These researchers reasoned that glutamate removal from the synaptic space by astrocyte-resident excitatory amino acid transporter-2 (EAAT2) is compromised in a PD mouse model induced by 1-methyl-4-phenyl-1,2,3,6-tetrahydropyridine (MPTP), which would result in continuous depolarization of the postsynaptic neuron and influx of copious amounts of Ca^2+^ into the presynaptic neuron that leads to oxidative stress. Further, the authors hypothesized that short-chain fatty acids (SCFAs), microbial metabolites produced by the gut microbiome, particularly propionic acid and butyric acid, would protect EAAT2 from oxidative dysfunction and thereby lead to decreased oxidative stress in this MPTP-initiated PD mouse model.

This overall hypothesis being investigated by the authors is consistent with oxidative modification and dysfunction of EAAT2 and subsequent oxidative damage in brains of individuals with another neurodegenerative disorder, Alzheimer disease [33].

Professor Liu and team demonstrated convincingly that, in contrast to results in control mice treated with MPTP, addition of SCFAs to this MPTP-initiated PD mouse model significantly prevented loss of tyrosine hydrolase (TH)-positive dopaminergic neurons in the substantia nigra and improved locomotion, motor, and muscle strength skills. Likewise, elevated levels of glutathione, increased expression of nuclear factor-erythroid 2-related factor 2 (Nrf2)-dependent antioxidant enzymes, as well as elevated levels of EAAT2, together with decreased neuroinflammation due to inflammatory cytokines associated with astrocyte activation, were observed in the SCFA-treated PD mouse model brains.

Inhibition of EAAT2 by dihydrokainic acid prevented these protective properties of SCFA-treated MPTP-initiated PD mice, strongly supporting the notion that inhibition of EAAT2 and consequent decreased removal of glutamate from the synapse underlies the pathological, behavioral and oxidative stress observed in this PD mouse model.

The authors conclude that the beneficial effects of SCFAs are mediated, at least in part, via the protection of EAAT2 and propose this key transport protein as a promising therapeutic target for PD.

#### 2.2.4. Microbial Metabolism of Levodopa as an Adjunct Therapeutic Target in Parkinson Disease

Professor Jimmy B. Feix and colleagues, in a Perspective Article, examine relatively recent investigations of the roles of microbial metabolism of the major drug used for the treatment of Parkinson disease (PD), L-3,4-dihydroxyphenylalanine (L-DOPA) and offer alternative approaches to enhance L-DOPA levels and effectiveness in PD over its long-term usage [Contribution 7].

As PD progresses in humans, L-DOPA therapeutic dose increases, often leading to development of L-DOPA-induced dyskinesia (LID). Gut bacteria in PD are different than in normal controls. Further, bacterial overgrowth in the jejunum, a major small-colon site for L-DOPA absorption, likely contributes to the metabolism of this drug primarily by *E. faecalis*, eventually leading to tyramine. The effect of this metabolism is lower levels of L-DOPA in the bloodstream, and therefore the brain. Higher doses of L-DOPA are needed for PD patients to try to overcome this jejunum-located bacterial overgrowth, further contributing to LID.

In an attempt to overcome this bacterial-mediated decarboxylation of L-DOPA by L-amino acid decarboxylase (AADC), an inhibitor of this latter enzyme (carbidopa) is often prescribed for PD patients. However, L-DOPA metabolism in *E. faecalis* in cultures was unaffected by carbidopa.

Accordingly, the authors investigated alternative approaches to achieve this aim. They took advantage of their prior work with mitochondria-directed drugs (MTDs), and noting that bacteria, like mitochondria, have a large internal negative transmembrane potential, these scientists prepared and tested a number of MTDs containing polyethylene glycol of various lengths for solubility and a triphenylphosphonium (TTP^+^) component to drive the drugs into bacteria. Substitution of three hydrogen atoms on these compounds with deuterium allowed for HPLC-mass spectrometry monitoring of these agents.

A small number of these drugs were tested in vivo in mice with exciting results: the deuterated drugs were highly successful in being absorbed in gut-homogenized samples and in inhibiting L-DOPA metabolism with little to no dopamine-d3 formed. In contrast, in brain isolated from these drug-treated mice, levels of dopamine-d3 were 15-fold higher than in control mice.

The authors assert with good evidence that these drugs can inhibit L-DOPA decarboxylation in the gut, which increased the amount of L-DOPA in the brain that can be converted by L-DOPA decarboxylase to dopamine.

Professor Feix and colleagues opine that the transient inhibition of *E. faecalis* in the gut appears to be a novel strategy to block L-DOPA metabolism in the intestine, without changing the microbiome composition. Therefore, these authors appear to have developed a novel therapeutic strategy for treatment of patients with PD. Of course, further rigorous studies will be required to confirm this novel approach in this disorder, but these preliminary results appear quite promising.

#### 2.2.5. Translational Feasibility of Curcumin for Treatment of Alzheimer Disease: A Critical Appraisal of Clinical Challenges

Professor Ralph N. Martins, Dr. Jasmine Priya Virk and colleagues provide a comprehensive review and critical assessment of the therapeutic potential of curcumin for the treatment of Alzheimer disease (AD) [Contribution 8].

Curcumin is obtained from the spice turmeric. As described by the authors, in in vitro studies, curcumin binds to amyloid beta peptide (Aβ42) when in small oligomeric form, thereby preventing or significantly modulating this neurotoxic peptide from being associated with oxidative stress. In addition, curcumin provides reduction in neuroinflammation in in vitro studies.

In contrast, the authors make strong statements about the lack of effectiveness of curcumin as an in vivo treatment for AD patients. The authors provide in Table 1 of their paper a helpful tabulation of the in vivo clinical studies in which curcumin either alone or with adjuvant moieties is given to human controls and AD patients. A short summary of this table suggests that curcumin treatment: (1) does not appear to result in clearance of Aβ or phosphorylated tau from brains; (2) does not support the notion that this treatment directly affects AD pathology or potential indirect means of affecting AD pathology; (3) does not decrease oxidative damage in brain; (4) lacks consistency in measures of neuroinflammation and new, more extensive clinical trials will be needed to definitively determine anti-inflammatory effects of curcumin; (5) provides some evidence of neurogenesis and increased neuronal spine density, the authors of the present review note different studies have different potential confounding variables and the authors suggest additional studies are needed to clarify the current literature; and (6) does not provide convincing clinical evidence for cognitive benefit.

Overall, the authors indicate that the lack of effectiveness of curcumin in in vivo studies related to AD treatment derives from several sources, among which are: (1) the poor solubility and resulting poor bioavailability of curcumin; (2) the nearly unambiguous finding that curcumin cannot reliably enter the brain parenchyma by crossing the blood–brain barrier; and (3) the rapid systemic metabolism of curcumin, which quickly lowers levels of this putative therapeutic for AD.

The authors of this critical appraisal for use of curcumin in the treatment of AD conclude their paper by stating, “the narrative of curcumin as a promising multi-target therapeutic for Alzheimer’s disease is compelling in theory but remains insufficiently supported by robust clinical evidence” and again, “while the curcumin saga offers valuable lessons in drug development and the perils of extrapolating from simplistic models, the evidence points to the field moving beyond the parent compound to more promising avenues.”

#### 2.2.6. The Heme Oxygenase/Biliverdin Reductase System as a Therapeutic Target to Counteract Cellular Senescence in Alzheimer’s Disease

For this Special Issue of *Antioxidants*, entitled “Oxidative Stress and Its Mitigation in Neurodegenerative Disorders,” Professor Cesare Mancuso provided a review article that focused on the heme oxygenase (HO-1, inducible form; HO-2, constitutive form)/biliverdin reductase (BVR-A) system and its involvement in cellular senescence [Contribution 9]. Professor Mancuso points out some of the normal functions of HO-1 and BVR-A. HO-1 degrades free heme, which otherwise would serve as a free radical-generation source, and the products of this reaction are biliverdin, carbon monoxide (CO) and ferrous iron (Fe^2+^). The author notes that in Alzheimer disease (AD) and its earlier stage, mild cognitive impairment (MCI), both HO-1 and BVR-A are oxidatively modified, with BVR-A mostly damaged by the excess presence of 3-nitrotyrosine [21,22,23].

The author provides the reader with a thorough background on senescent cells, including their formation and the damage to other cells caused by such senescent cells. Included in this discussion is his description of the early phase of cell senescence as an adaptive cellular response to decrease excess heme through the activity of HO-1. He indicates that this adaptive response would be compromised if there were insufficient levels of iron-binding proteins such as ferritin. As is well-known, Fe^2+^ reacts with abundant hydrogen peroxide in cells to produce, by Fenton chemistry, the highly reactive and damaging hydroxyl free radical. Therefore, sufficient levels of ferritin are necessary to bind ferrous iron to keep it from participating in Fenton chemistry and damaging cells, reducing inflammatory responses and other deleterious reactions.

As discussed in this review paper, BVR-A is well known in AD and MCI brains for its involvement in disruption of insulin signaling and insulin resistance [34], with nitrosative modification of key tyrosine residues on BVR-A [1,21].

The author concludes this review paper with a description of his hypothesis that modulation of cellular senescence by the HO-1/BVR-A system potentially will enhance the availability of therapeutic targets for AD. Among these potentially useful agents for affecting the HO-1/BVR system are:

(1) The acetylcholine esterase inhibitor, donepezil, combined with a non-competitive NMDA receptor inhibitor, memantine, delivered subcutaneously in the SAMP8 mouse model of AD, lowered staining of an Aβ-produced marker of AD in hippocampal CA1 and CA3 regions.
(2) A 14.5-month-long treatment with a statin (atorvastatin) in aged beagle dogs, who have an identical amino acid sequence of Aβ42 to that of humans, led to increased parietal levels and activity of the HO-1/BVR-A system along with a marked decrease in oxidative and nitrosative stress compared to aged beagle dogs not treated with atorvastatin. Such treated dogs had improved memory and decreased BACE1 activity, i.e., less Aβ42 production [35], consistent with the finding that in a retrospective analysis of studies involving over 6 million patients, statin treatment was associated with lower AD occurrence in an age-dependent manner [36].
(3) Studies with NSAIDS, in aggregate, do not seem to be highly effective in modulating AD.
(4) Rapamycin, an mTORC1 inhibitor that prevents loss of autophagy, also elevates HO-1 expression, lessens lipid peroxidation and DNA oxidation in Zn^2+^-treated rats, suggesting a possible involvement of HO-1, but the evidence is not particularly strong.
(5) Ferulic acid (FA), a polyphenol found in many fruits and vegetables, and especially the ethyl ester of ferulic acid (FAEE), the latter more able to cross the BBB, given intraperitoneally to gerbils, prevented Aβ42-mediated protein oxidation and lipid peroxidation in isolated synaptic membranes, accompanied by elevation of HO-1 levels [37].

Cellular senescence and its suppression by the HO-1/BVR-A system is in its infancy of study and requires more to learn, but, in this Guest Editor’s opinion, this Special Issue review by Professor Mancuso is a good beginning.

#### 2.2.7. Natural Vitamins and Novel Synthetic Antioxidants Targeting Mitochondria in Cognitive Health: A Scoping Review of In Vivo Evidence

Professor Malika G. Fernando and colleagues, including Professor Ralph N. Martins, performed a literature review of certain vitamins and novel mitochondria-targeting drugs [Contribution 10]. Three major databases were employed: MEDLINE, EMBASE and Scopus. The papers examined involved in vivo models of Alzheimer disease (AD), dementia, cognitive decline, aging, oxidative stress or mitochondrial dysfunction that, in each case, had been treated with either natural vitamin antioxidants or mitochondria-targeted antioxidants. Only articles written in English and dealing with in vivo studies, specified antioxidants, and the presence of dementia, cognitive issues or mitochondrial health were included. Exclusion criteria included non-English language use, multivitamin supplementation, vitamin precursors or irrelevant outcomes.

The authors identified 13 papers involving natural vitamin antioxidants (NVAs) and 12 papers involving mitochondria-targeted antioxidants (MTAs) that met the inclusion criteria from 768 publications examined. Table 1 in the paper is helpful to the reader in summarizing the results of this paper.

Vitamins, including vitamin E, retinoic acid, methylcobalamin (vitamin B12), folate (vitamin B9), niacin (vitamin B3), thiamine (vitamin B1) and pantothenic acid (vitamin B5), were each reported to have positive effects in mostly mouse models of AD. However, as reported by the authors of the current review, clinical trials in humans with cognitive decline employing vitamins E, C, A, and vitamin B-complex yielded mixed or marginal results.

MTAs, including MitoQ, MitoTempo, SS-31 (Elamipretide), and SkQ1 (plastoquinone decyltriphenylphosphonium), are positively charged compounds directed to mitochondria via the latter’s large negative membrane potential. In preclinical studies, each improved important mitochondrial properties, among which, depending on the MTA and the in vivo model used in the respective study, increased cognitive performance, increased lifespan or protection of the mitochondrial electron transport complexes I and IV, reduced hippocampal and cortical cell damage, reduced oxidative stress in the brain, improved synaptic function, and improved hippocampal function. However, though compared to NVAs, MTAs had consistently better and reproducible performance in preclinical studies, the authors of the current review indicate there have been no reported clinical studies on these compounds. The authors recommend that future research should study the efficacy of MTAs at various levels of cognitive loss, including dementia, and various stages of AD.

As a note from the author of this Editorial of this Special Issue of *Antioxidants*, I would recommend readers examine the studies of Prof. Gary E. Gibson, including his several papers on benfotiamine, one with positive results of a Phase IIa clinical trial of this drug with MCI patients [38].

#### 2.2.8. Ginkgo Biloba Extract Ameliorates Age-Related Mitochondrial Deficits in Human iPSCs and Their Derived Neurons and Astrocytes

Prof. Anne Eckert and her colleagues investigated whether and how ginkgo biloba extracts (GBE) protected against deficits in age-related mitochondrial deficits in induced pluripotent stem cells (iPSCs) and their differentiated neurons and astrocytes relative to similar young moieties [Contribution 11].

These researchers confirmed that, relative to iPSCs from young donors and differentiated neurons, iPSCs from aged donors and their differentiated neurons had altered mitochondrial properties, including decreased ATP production and mitochondrial membrane potential, but increased mitochondrial reactive oxygen species (mtROS) and superoxide free radical levels. The authors assert that, for the first time, astrocytes formed from differentiated iPSCs from aged donors had similar mitochondrial alterations compared to astrocytes derived from iPSCs from young donors.

When GBE was added for 24 h to iPSCs, several important results compared to lack of GBE treatment were obtained: (1) mitochondrial properties in both young and aged iPSCs were improved, while oxidative stress was significantly lessened in these cells; (2) when subjected to Seahorse analysis, the oxygen consumption rate (measure of ATP formation) and the extracellular acidification rate (index of glycolytic rate) were shown to be elevated in iPSCs from both aged and young donors; and (3) GBE treatment of aged-derived iPSC-differentiated neurons and astrocytes led to increased mitochondrial function and decreased oxidative stress.

The study discussed here could not determine mechanisms for the observed results, but in the opinion of the Guest Editor of this Special Issue of *Antioxidants*, the results do support the notion that further studies with GBE are warranted. The authors outline a number of possibilities for such future studies, which, if successful, will be highly illuminating for astrocyte-neuronal interactions in brain mitochondrial function.

The authors outline some limitations of the study, including the following: (1) GBE is composed of several kinds of molecules, which will be important to dissect to determine the most beneficial one(s). (2) This study was ex vitro in nature. Future studies will be necessary to determine if the positive effect observed in iPSCs and their derived neurons and astrocytes in cultures is also observed in in vivo studies.

#### 2.2.9. Cannabinol-Facilitated Modulation of Genes Involved in Oxidative Stress Response and Neuronal Plasticity: A Transcriptomic Analysis

Prof. Emanuela Mazzon and colleagues investigated the dose–response of cannabinol (CBN) on neuroblastoma cells fused with motor neuron-enriched embryonic spinal cord (NSC-34) cells using transcriptomics [Contribution 12]. The fusion of these two cell types produces neuronal biology. CBN is a relatively minor cannabinoid derived from D^9^-tetrahydrocannabinol (D^9^-THC). This study was also designed to test if CBN could exert similar effects as major components of the former.

Transcriptomics led to the overexpression of genes for 15 pathways, from which three were investigated further: Cellular Responses to Stimuli; Cellular Responses to Stress; and Axonal Guidance. Two of the major findings of this study using CBN on NSC-34 cells were as follows: (1) At any concentration of CBN used, no adverse effects on the NSC-34 cells were observed, including the downregulation of genes associated with apoptotic cell death (*caspase 3*), in contrast to the effects of THC. (2) CBN was able to induce protective pathway genes related to decreasing the effects of oxidative stress, i.e., activation of genes leading to Nrf-2 and its cellular stress response to produce antioxidant enzyme genes, while downregulating genes for the Nrf-2 inhibitor, Keap1. (3) Additionally, the gene for Cu,Zn-superoxide dismutase (*Sod1*) was downregulated, which would lead to less hydrogen peroxide production, a known precursor to elevated oxidative stress. Overexpression of SOD1 protein is a known player in oxidative stress in the neurodegenerative disorder, ALS.

The researchers found evidence for activation of the Akt pathway, as the increase in phosphorylated Akt/Akt increased with CBN dosage. These scientists noted that the pAkt/Akt ratio is implicated in neuronal polarization and dendritic branching.

The authors seem to promote the potential use of CBN for in vivo studies to determine if this agent is as active in living species as in cellular disease models.

#### 2.2.10. Protective Functions of β-Alanyl-L-Histidine and Glycyl-L-Gylcoconjugates and Copper in Concert

Professor Enrico Rizzarelli and his colleague, Dr. Irina Naletova, provide an in-depth overview of Cu-bound peptide redox chemistry, in which the scientists extensively review the protective aspects of the peptides β-alanyl-L-histidine, aka carnosine (Car) and glycyl-L-histidinyl-L-lysine (GHK) conjugated to a disaccharide or a polysaccharide, respectively [Contribution 13].

The non-reducing disaccharide trehalose (Tre, α-D-glucopyranosyl-(1 → 1)-α-D-glucopyranoside) bound to Car to form CarTre, or the polysaccharide hyaluronan, a polymer of various subunit lengths of hyaluronic acid (HA, β-1,4-D-glucuronic acid and β-1,3-N-acetyl-D-glucosamine) bound to GHK to form HAGHK and to Car to form HACar are reviewed as potent antioxidants against hydroxyl free radical damage and to binding of oxidative stress-derived products, for example in the case of CarTre to acrolein. The authors note that HA protects Car from the latter’s degrading enzyme, carnosinase.

The copper complexes of HACar derivatives are described by the authors to have substantial SOD1-like superoxide free radical scavenging properties, while HAGHK polymers have scavenging properties against ROS. These Car- and GHK-glycoconjugates are also described as being beneficial in preventing damage associated with Aβ peptide or participating in the dissolution of Aβ plaques.

The authors opine that further investigations of Car and GHK, as well as their respective bound glycoconjugates, for in vivo studies of neurodegenerative disease models are warranted.

## 3. Conclusions

The articles assembled in this Special Issue of *Antioxidants* illustrate a primary role of oxidative stress and/or its mitigation in neurodegenerative disorders. Oxidative and/or nitrosative damage to proteins, membrane lipid bilayers and DNA/RNA leads to conformational alterations that, in turn, impair their respective functions [1,2]. This oxidative- and/or nitrosative-stress damage and consequent loss of function often lead to death of key brain cells and are critical aspects of the etiology, pathology and progression of neurodegenerative disorders [1,2,3].

Mitigation of oxidative- and/or nitrosative stress-induced damage to key cellular components in the brain is a subject of significant inquiry in neurodegenerative disorders. However, the experiences with the success of antioxidant vitamins in cellular models of Alzheimer disease or Parkinson disease have been largely unsuccessful in *in vivo* studies of patients in these two most affected populations of age-related neurodegenerative disorders. A caveat to the above may be the recent results of a Phase IIa clinical trial in AD in which a vitamin B1 derivative has shown some success [38].

Consequently, investigators, including some of those included in this Special Issue, have looked for alternatives for prevention or at least to ameliorate the effects of oxidative damage and brain cell death. Some quite promising approaches are described in this Special Issue. Clearly, additional studies will be required, but the critical role of oxidative stress in the underlying aspects of neurodegenerative disorders, although well-established, requires additional attention to gain further insights into the etiology, pathology, and progression of these disorders.

## Data Availability

No new data were created or analyzed in this study. Data sharing is not applicable to this article.

## Abbreviations

The following abbreviations are employed in this manuscript:

AADC	L-Amino acid decarboxylase
Aβ42	Amyloid beta-peptide composed of 42 amino acids
AD	Alzheimer disease
AGEs	Advanced glycation end products
ALS	Amyotrophic lateral sclerosis
ARG	Arginine
BER	Base excision repair
BVR-A	Biliverdin reductase-A
Car	β-Alanyl-L-histidine, aka carnosine
CBN	Cannabinol
CO	Carbon monoxide
CK	Cockayne syndrome
CYS	Cysteine
D^9^-THC	D^9^-tetrahydrocannabinol
L-DOPA	L-3,4-dihydrophenylalanine
DS	Down syndrome
EAAT2	Excitatory amino acid transporter-2
ER	Endoplasmic reticulum
ET	Ergothioneine
FA	Ferulic acid
FAEE	Ferulic acid ethyl ester
GBE	Ginkgo biloba extract
GHK	Glycyl-L-histidinyl-L-lysine
HA	Hyaluronic acid: β-1,4,-D-glucuronic acid and β-1,3-N-acetyl-D-glucosamine
HF	Heart failure
HNE	4-hydroxynonenal
HO-1	Heme oxygenase-1
HO-2	Heme oxygenase-2
iPSCs	Induced pluripotent stem cells
LID	L-DOPA-induced dyskinesia
MCI	Mild cognitive impairment
MPTP	1-Methyl-4-phenyl-1,2,3,6-tetrahydropyridine
MTAs	Mitochondria-targeted antioxidants
MTDs	Mitochondria-directed drugs
MtROS	Mitochondrial reactive oxygen species
NER	Nucleotide excision repair
Nrf-2	Nuclear factor-erythroid 2-related factor 2
NSC-34	Neuron-enriched embryonic spinal cord
NVAs	Natural vitamin antioxidants
OCTN1	Organic cation transporter novel type 1
PD	Parkinson disease
PERK	Protein kinase R-like ER kinase
RAGE	Receptor for advanced glycation end products
ROS	Reactive oxygen species
SCFAs	Short-chain fatty acids
SkQ1	Plastoquinine decyltriphosphonium
SS-31	Elamipretide
SOD1	Superoxide dismutase-1
TH	Tyrosine hydrolase
TMA	Trimethylamine
TMAO	Trimethylamine-N-oxide
Tre	α-D-glucopyranosyl-(1 → 1)-α-D-glucopyranosideaka trehalose
XP	Xeroderma pigmentosum
